# Personal values and people’s attitudes toward older adults

**DOI:** 10.1371/journal.pone.0288589

**Published:** 2023-08-02

**Authors:** Joelle H. Fong, Ting-Yan Wang

**Affiliations:** Lee Kuan Yew School of Public Policy, National University of Singapore, Singapore, Singapore; University of Baltistan, PAKISTAN

## Abstract

**Background:**

We examine the relationship between people’s personal values and their attitudes toward older adults. In addition to the two conventionally-used measures of personal values (agency subdimension and communion subdimension), we distinguish across 10 different value types and explore how each impacts attitude.

**Methods:**

We use data from the World Values Survey for three aging Asian societies, namely Japan (*N* = 2448), Singapore (*N* = 1972), and Hong Kong PRC (*N* = 1000). For each sample, we perform regression-based analyses to assess the relative importance of the 10 value types in explaining people’s attitudes towards older adults. Results are then compared against regressions based on the two aggregate value measures.

**Results:**

In all three economies, the agency subdimension was a more consistent predictor of unfavorable attitudes toward older adults, as compared to the communion subdimension. Our disaggregated analysis reveals two additional insights. First, the positive association between agentic values and attitudes was driven predominantly by the power (wealth) and stimulation (excitement) value types. Second, the lack of association between the communion subdimension and attitudes must be interpreted with caution since certain value types within this subdimension may act in opposite directions causing effects to cancel each other out at the aggregate level.

**Conclusions:**

Disaggregating personal value types provides greater prognostic power than the two aggregate measures, as well as insights on ways to improve people’s attitudes toward older adults. Interventions aimed at reducing ageist attitudes in aging societies can target individuals with agentic traits by emphasizing notions of power (e.g., older adults’ economic success) and stimulation (e.g., positive images of older adults learning new things).

## Introduction

Global population ageing has increased policy attention on how societies view, treat and accommodate the growing numbers of older people around the world. An extensive literature has shown that people often hold more negative attitudes toward older adults than younger adults in many societies [1–4]. Such negative attitudes may stem from views that older persons are mentally slower, forgetful, ill, a burden to society, less competent, and unproductive [3, 5], as well as concerns of shifts in government spending favoring older persons in ageing societies [6]. Researchers have sought to better understand the extent of these biases towards older people, and importantly, what factors critically influence such attitudes. For example, some studies investigate the extent of ageism in different aging populations [6, 7], as well as how intergenerational contact can improve attitudes towards older people [8, 9]. A number of studies also demonstrate that individual traits such as gender, age, education, and religious beliefs are strongly associated with people’s attitudes toward older adults [6, 10–12].

A growing body of work has highlighted the importance of personal values as a predictor of peoples’ attitudes towards older adults even in the presence of individual-level characteristics like age and gender [11–13]. Values have been described as goals that specify what is important and meaningful in life, as well as cognitive representations of basic motives that are important to individuals in their lives and guide their perception, judgments, attitudes, and behaviors [14, 15]. These basic values are “motivationally distinct” in that they are differentiated by their underlying goal [16], but can also be grouped or clustered into two broad dimensions based on the distinction between agency (dominance, power, status) and communion (friendliness, warmth, love) [17–21]. The agency subdimension (or agentic values) comprise various basic values and qualities relevant for goal attainment, striving for mastery, and social dominance. The communion subdimension (or communal values) comprise basic values relevant for cooperation, maintenance of social relationship, and the fulfilment of social bonds.

Past studies have collectively demonstrated that agentic and communal values shape peoples’ attitudes towards older adults. However, empirical findings from the literature are mixed regarding the respective roles of the two subdimensions. For instance, one study covering more than 30 economies across various cultures found that only the communion subdimension was significantly associated with positive views of older adults. There was no association between the agency subdimension and attitudes in the meta-analysis [12]. By contrast, another study reported that only the agency subdimension was predictive of attitudes toward older adults, whereas the communion subdimension was not [13] The authors attributed the significance of agentic values to increased concerns about resource scarcity and competition across generations in rapidly aging societies, especially among younger adults who have become more critical of collective welfare schemes over time. Yet, at the same time, one may also expect communal values to be associated with attitudes since communion is other-oriented and closely related to the liking of others [22, 23]. In sum, it is unclear from the extant literature why only the agency subdimension or the communion subdimension—but not both—explains individuals’ attitudes toward older adults.

In this present study, we explore a disaggregated approach to examining the relationship between personal values and people’s attitudes toward older adults. Using Schwartz’s theory of values, we distinguish across 10 different value types and perform regression-based analyses to assess their relative importance in explaining attitudes towards older adults. We contend that looking beyond the (aggregate-level) agency and communion subdimensions will provide new insights to help policymakers and administrators better understand the relationship between individuals’ personal values and their attitudes toward older adults. Also, given that the 10 basic values are “motivationally distinct” [16], it is possible that certain value types are more strongly associated with attitudes than others. This may have implications for interventions aimed at improving negative and stereotypical views of persons in older ages. By clarifying the relative importance of the 10 different value types in explaining people’s attitudes toward older adults, we also seek to reconcile the mixed findings from past studies that have focused exclusively on the aggregate-level subdimensions.

## Context and literature review

### 10 basic values and the two subdimensions

Values have been described as goals that specify what is important and meaningful in life [14, 15]. The conceptual framework for the present research stems from Schwartz’s theory of basic values which outlines 10 basic personal values derived from the universal requirements of human life, namely, Power, Achievement, Hedonism, Stimulation, Self-Direction, Universalism, Benevolence, Tradition, Conformity, and Security [14, 24–26]. Importantly, the 10 basic values are “motivationally distinct” because they are differentiated by their underlying goal or motivation [16]. These values are also universal in that they help humans cope with one or more of universal requirements of existence (e.g. social interaction, individual needs), and thus the theory “concerns the basic values that people in all cultures recognize” [16]. These basic values are not only linked to one’s personality and self-concept, but also connected to the socialization process. Schwartz’s theory has been validated in many countries, including Asian economies such as Singapore, China, and Malaysia [16, 27, 28].

Agency and communion provide a superordinate framework of organization for more granular taxonomies of goals and values [20, 29]. The distinction between agency and communion is among the most influential pairings of content in psychology and behavioural sciences. In framing his socio-analytic theory around the distinction of agency and communion, Hogan [18] succinctly labelled the set of agentic values as “getting ahead” and the set of communal values as “getting along”. Thus, theoretically and in practice, the 10 basic values can be grouped into two aggregate-level subdimensions. A typical classification method used in past research [13, 30] is as follows: Power, Achievement, Hedonism, Stimulation, and Self-Direction are grouped under the agency subdimension, while Universalism, Benevolence, Tradition, Conformity, and Security are grouped under the communion subdimension. Each subdimension comprise of five component value types and their scores are linearly combined to derive an aggregate score respectively for the set of agentic values and the set of communal values.

### Known determinants of attitudes toward older adults

In this sub-section, we review past studies which have examined determinants of peoples’ attitudes toward older adults. This provides basis for our selection of covariates in subsequent empirical analysis. Aside from personal values, individual traits that have been shown to influence their attitudes toward older adults include gender, age, education, income, religious beliefs, and post-materialism. Women were less likely than men to hold negative views of older adults in various societies, in part due to their personal experiences in family care [11, 13]. The literature is mixed on the association between age and perceptions of older adults. While some studies document that younger adults were less likely to subscribe to age-stereotype-consistent attributions or view seniors as a burden to society [10, 13], others showed that negative attitudes toward older persons tends to decrease with age [11, 12].

Studies have further documented that highly educated respondents were less likely to have negative views of the older population, controlling for other confounders [11–13]. This effect was independent from age, implying that educational policy and practice could be a means to help reduce negative views of older adults. Income and stronger religious beliefs were positively correlated with positive attitudes toward older adults [12]. By contrast, Peterson and Ralston [11] found that religiosity/being religious increased the odds of viewing older persons as a burden. Post-materialism—a value orientation emphasizes self-expression and quality of life over economic and physical security—is likely to engender more positive views of older persons [6]. While some studies found partial support for this [13], others found evidence to the contrary [11].

### Geographical context

So far, such effects of personal traits on attitudes toward older adults have mostly been studied in Western contexts, primarily Europe and North America. The present study extends this work to the Asian Confucian context by analyzing data from three Asian societies. This is important and timely since the older populations in East Asia and the Pacific is growing much faster than in other regions. By 2050, nearly two-thirds of the world’s older people will be living in Asia-Pacific [31]. We use representative community-based samples from Singapore, Hong Kong PRC, and Japan. Japan became a “super-aged” country in 2006, where more than one in five of the population is 65 or older [32]. Singapore and Hong Kong PRC are amongst the most rapidly aging economies in Asia-Pacific and are projected to become super-aged societies by around 2025. In addition, the three societies that we selected fundamentally share traditional and communitarian values stemming from Confucianism as a system of social and ethical philosophy. This allows for comparability across the country-specific analysis. Our research findings are particularly relevant to help inform the design of policy interventions towards improving ageist attitudes in these aging societies.

## Materials and methods

### Data

Data was sourced from the sixth wave of the World Values Survey (WVS). WVS is one of the largest non-commercial panel datasets of public attitudes and values globally, covering almost 100 countries [33]. Survey questionnaires adopted in each country were drawn from the standard items proposed by WVS. A stratified sampling strategy was adopted in Singapore and Hong Kong PRC, while a combined method of random sampling and quota sampling was employed in Japan. Face-to-face interviews were conducted using computer-assisted techniques or hardcopy questionnaires. Community-dwelling persons age 18 and older were interviewed (*N* = 1,972 (Singapore); 1,000 (Hong Kong, China), and 2,448 (Japan)). To derive representative population samples, we applied the country-specific individual-level weights in all analyses. These weights serve to compensate for small deviations in the resulting sample with respect to sex-age distribution or rural-urban distribution that are considered important to obtain reliable results [33].

### Dependent variables

WVS respondents were asked: “Now could you tell me whether you agree, agree strongly, disagree or disagree strongly with each of the following statements?”, where statement (1) is “Older people are a burden on society” and statement (2) is “Older people get more than their fair share from the government.” An indicator variable was constructed for each item by combining “agree strongly” and “agree” into a single category (coded as 1) to indicate a negative attitude toward older persons. The reference category included “disagree” and “strongly disagree” responses (coded as 0). Because the item descriptions are negatively-worded, these variables measure ageism-related attitudes to some extent [34]. Respondents with missing data for these outcome variables were excluded from the regression analyses. Percentage of missings was <1% for Singapore and Hong Kong PRC. Japan had a higher percentage of missings but its base sample was much larger than the other two economies, so the analytical samples were still relatively comparable.

### Key explanatory variables

Table 1 shows the definitions of the 10 value types as adapted from Schwartz [24], alongside the corresponding survey questions. The WVS question is phrased as follows: “Now I will briefly describe some people. Would you please indicate for each [*description*] whether that person is very much like you, like you, somewhat like you, a little like you, not like you, or not at all like you?” For instance, the description outlined for Power value type is “It is important for this person to be rich” while that for Security value type is “It is important for this person living in secure surroundings”. Responses ranged from 1 (“not very like me”) to 6 (“very much like me”). We constructed 10 continuous variables—a higher score indicating greater alignment with a given value type. Next, an aggregate subdimension score for agentic values was obtained by linearly combining the Power, Achievement, Hedonism, Stimulation, and Self-Direction scores. Scores for Universalism, Benevolence, Tradition, Conformity, and Security were aggregated to derive a communal values subdimension score. Each subdimension score also ranged from 1 to 6.

**Table 1:** 10 motivationally distinct value types in Schwartz’s theory and WVS question wordings.

s/n	Personal values and dimensions	Definition	WVS question wording (*Now I will briefly describe some people*. *Would you please indicate for each description whether that person is very much like you*, *like you*, *somewhat like you*, *a little like you*, *not like you*, *or not at all like you?……)*
	Agentic values (greater self focus):		
1	Power	Social status and prestige, control or dominance over people and resources	It is important for this person to be rich
2	Achievement	Personal success through demonstrating competence according to social standards	It is important for this person being very successful
3	Hedonism	Pleasure and sensuous gratification for oneself	It is important for this person to have a good time
4	Stimulation	Excitement, novelty, and challenge in life	It is important for this person to adventure and taking risks
5	Self-direction	Independent thought and action in choosing, creating, exploring	It is important for this person to think up new ideas and be creative
	Communal values (greater social focus):		
6	Security	Safety, harmony, and stability of society of relationship, and of self	It is important for this person living in secure surroundings
7	Conformity	Restraint of actions, inclinations, and impulses likely to upset or harm others and violate social expectations or norms	It is important for this person to always behave properly
8	Tradition	Respect, commitment, and acceptance of the customs and ideas that traditional culture or religion provide	It is important for this person tradition
9	Universalism	Understanding, appreciation, tolerance, and protection for the welfare of all people and for nature	It is important for this person to looking after the environment
10	Benevolence	Preservation and enhancement of the welfare of people with whom one is in contact with	It is important for this person to help the people nearby

Sources: Definitions adapted from Schwartz (1994) and survey question wordings from WVS (2021).

Notes: WVS = World Value Survey.

### Control variables

Following prior studies, age, sex, marital status, income, education, religiosity, and post-materialism were included as control variables. Respondents were grouped into five age categories including 18–30, 31–40, 41–50, 51–60, and 60+ (reference). An indicator variable was used for sex (1 = female, 0 = male). Categories for marital status were single (reference), married/cohabiting, others (divorced, widowed, or separated). Both income and education were treated as continuous variables. Income was measured on a 10-point scale, with 10 being the highest income group. Educational attainment was measured on a 9-point scale ranging from 1 (no formal education) to 9 (university-level education). We also included an indicator variable for religiosity set to 1 if the respondent stated he or she is a religious person. The post-materialist values score, based on Inglehart’s classic 12-item index, was available in the WVS data. It ranged from 0 to 5, with a higher score indicating more post-materialist values.

Drawing on the rich dataset, we considered four additional confounders. The first was whether currently employed (1 = yes, 0 = no) since participation in the workforce may independently influence attitudes towards older persons even in the presence of socio-economic factors. People who are more satisfied with their own life are generally likely to view situations and others more positively [35]. Thus, we included trust in one’s family (range 1–4) and level of life satisfaction (range 1–10). Finally, we included beliefs about government responsibility measured using respondents’ extent of agreement with the statement that “government should take more responsibility to ensure that everyone is provided for” (continuous variable ranging 1–10). Those who agree with this statement about greater government responsibility and care for older persons may be less inclined to perceive older adults negatively.

### Statistical analyses

Our descriptive analysis began with a comparison of the mean scores (or prevalence) of the 10 different value types across the three samples. This breakdown is valuable since certain personal values may be more prevalent in one society as compared to others, even if the aggregate-level agentic/ communal scores appear to be relatively similar. Reliability tests were conducted to verify that, for each sample, these distinct value types can be meaningfully clustered into two aggregate subdimensions (communal versus agentic). A generally accepted rule is that Cronbach’s α value of 0.60 or higher indicates an acceptable level of reliability. We then reported the mean scores for the aggregated measures, alongside the sample characteristics.

Separate regression analyses were conducted for the two outcomes: “burden on society” and “get more than their fair share”. We implemented hierarchical logistic regressions using first the aggregated value subdimensions, then the 10 different value types. For analysis using the aggregated measures, we specify three models: model 1 included only the two value measures, age, and sex. Model 2 added the standard control variables (e.g., marital status, income, and education), while Model 3 (final model) added more potential confounders as discussed above (e.g., employment, trust in family, and life satisfaction). This hierarchical approach provided a means to observe whether the effects of agentic and communion subdimensions on attitudes were robust to the additional of covariates in the model.

Next, we turned to the question of whether the disaggregating the personal value measures would be more useful and informative than the aggregate measures. That is, we replaced the two subdimensions with the 10 different value types in the final model (Model 3) and re-ran the regressions. To empirically evaluate whether this new set of results was more informative than the earlier version, we compared the pseudo R-squared values. Lastly, we conducted sensitivity analyses by excluding older persons aged above 60 from the sample. Odds ratios (ORs) and robust standard errors from the multiple logistic regression models are reported. All analyses were performed using STATA version 16.0 (STATA Corp., TX, USA).

## Results

### Prevalence of the 10 basic value types

Table 2 shows the mean score (prevalence) and standard deviation of each of the 10 personal value types by sample population. These scores reflect the sampled respondents’ extent of agreement with that particular value type. We see that in Hong Kong PRC and Japan, self-direction (preferring reliance on one’s own capacities) and hedonism (pursuit of gratification for oneself) were more salient than the other agentic-type values. Self-direction also ranked highly in Singapore, alongside achievement (personal success through demonstrating competence). Within the communal subdimension, higher mean scores were observed for security (social order, family security, national security) and benevolence (concern for those within the family and other primary groups) in Singapore and Hong Kong. In contrast, the Japanese resonated more strongly with universalism values (concern for the welfare of those in the larger society). Importantly, reliability checks confirmed the internal reliability of the items within the agentic subdimension, and separately, the items in the communal subdimension. Cronbach’s α values were consistently high across all three samples: 0.62–0.76 for agentic subdimension and 0.65–0.69 for communal subdimension.

**Table 2:** Mean scores (prevalence) of the 10 personal value types.

s/n	Personal values and dimensions	Singapore	Hong Kong	Japan
	Agentic values			
1	Power	3.59 (1.38)	2.93 (1.29)	2.07 (0.92)
2	Achievement	4.05 (1.22)	3.79 (1.29)	2.84 (1.17)
3	Hedonism	3.81 (1.29)	4.09 (1.28)	2.90 (1.13)
4	Stimulation	3.59 (1.31)	2.76 (1.38)	2.17 (1.02)
5	Self-direction	4.06 (1.31)	4.01 (1.28)	3.36 (1.21)
	Agentic values aggregate score	3.82 (0.93)	3.52 (0.85)	2.65 (0.70)
	*Cronbach’s alpha (α)*	*0*.*76*	*0*.*67*	*0*.*62*
	Communal values			
6	Security	4.46 (1.15)	4.44 (1.13)	3.63 (1.20)
7	Conformity	4.14 (1.13)	4.03 (1.27)	3.20 (1.11)
8	Tradition	4.13 (1.18)	3.85 (1.41)	2.89 (1.23)
9	Universalism	4.06 (1.09)	4.36 (1.13)	3.67 (1.06)
10	Benevolence	4.35 (1.10)	4.59 (0.99)	3.58 (1.08)
	Communal values aggregate score	4.23 (0.75)	4.25 (0.77)	3.39 (0.74)
	*Cronbach’s alpha (α)*	*0*.*69*	*0*.*65*	*0*.*65*

*Notes*: Data is from WVS study wave 6 (2010–2014). Means are shown for all grouped and disaggregated variables and standard deviations are in parentheses. Responses for each personal value question ranged from 1 (not at all like me) to 6 (very much like me). The two aggregate variables—agentic values and communal values—are derived based on a linear combination of the five indicators within each dimension. The Cronbach’s alpha value provides a measure of internal consistency, that is, how closely related a set of items are as a group. Individual-level weights are applied.

### Aggregate value subdimensions and other sample characteristics

In terms of attitudes towards older adults, 6.1% in Japan, 18.3% in Hong Kong PRC, and 16.3% in Singapore agreed with the first statement that “older people are a burden on society” (see Table 3). Larger proportions of participants agreed with the second statement that “older people get more than their fair share from the government”, with 40.9% in Japan, 52.4% in Hong Kong, and 44.3% in Singapore. Communal values were more dominant than agentic values in all three Asian societies. For instance, communal and agentic values scores averaged 4.23 and 3.82 respectively in Singapore, and 3.39 and 2.65 respectively in Japan. Post-materialist values were slightly more evident in Hong Kong than the other two economies. The socio-demographic characteristics were mostly comparable across the three weighted samples although, on average, Japan had a slightly older respondent profile. Japanese also had relatively stronger beliefs in government’s responsibility to provide for its citizenry. About 6 in 10 Singaporeans described themselves as religious, compared to 25.4% in Japan and 19.9% in Hong Kong.

**Table 3:** Sample characteristics and summary statistics.

Variables	Singapore		Hong Kong		Japan	
	Mean	SD	Mean	SD	Mean	SD
*Attitudes towards older persons*						
Older people as a burden on society	16.34%		18.25%		6.09%	
Older people get more than their fair share	44.26%		52.37%		40.85%	
*Personal values (aggregate)*						
Agentic values	3.82	0.93	3.52	0.85	2.65	0.70
Communal values	4.23	0.75	4.25	0.77	3.39	0.74
Post-materialist values	2.12	1.08	2.21	1.18	2.09	0.91
*Socio-demographic characteristics*						
Female	56.94%		54.50%		51.82%	
Age bands:						
18–30	25.71%		21.90%		13.59%	
31–40	15.62%		19.70%		17.44%	
41–50	19.18%		21.70%		16.41%	
51–60	18.55%		19.40%		18.95%	
Above 60	20.94%		17.30%		33.61%	
Marital status:						
Single	29.84%		29.90%		19.03%	
Married/ Cohabiting	65.02%		62.40%		69.95%	
Others (divorced, widowed)	5.14%		7.70%		11.01%	
Income	5.63	1.56	4.72	1.90	3.98	2.46
Education	5.31	2.55	5.88	2.31	6.68	1.81
Religious	60.26%		19.90%		25.42%	
Currently employed	54.31%		55.40%		63.20%	
Trust family	1.21	0.47	1.19	0.45	1.27	0.47
Satisfied with life	6.96	1.63	6.85	1.84	6.91	1.95
Importance of government responsibility	5.71	2.38	4.91	2.48	7.28	2.26
*N =*	*1972*		*1000*		*2443*	

*Notes*: Data is from WVS study wave 6 (2010–2014). Percentages shown for categorical variables; means and standard deviations shown for continuous variables. Individual-level weights are applied.

A breakdown of the key variables relating to attitudes and personal value, by age and by sex, is shown in the online supplementary material (S1 Table). We note that the mean values of both the agentic values measure and the communal values measure are relatively similar between males and females in each sample. However, larger proportions of males hold ageist-related attitudes than females in general. Comparing across age groups, we observe higher prevalence of persons above age 60 agreeing with the statement that older people are a burden on society, especially in Hong Kong PRC and Japan. About 33% of Hong Kongers above age 60 agreed with the *burden* statement (compare just 18% in the full sample). Nonetheless, the proportions of people who agreed with the *more than fair share* statement is rather uniform across age groups. There is also little variation in the mean values of the two aggregate personal values measures by age.

### Logistic regressions using aggregate subdimensions

The first three columns of Table 4 present the regression results for the dichotomous dependent variable that *older people are a burden*. Results indicated that the agentic values subdimension was significantly and positively associated with the *burden* view in all three societies in the final model, controlling for various confounders. The estimated OR for agentic values was 1.339 for Singapore, 1.881 for Hong Kong, and 1.958 for Japan, each statistically significant at the 0.1% level. This association was robust to the subsequent addition of control variables in the intermediate models; see online supplementary material (S2 and S3 Tables). In contrast, the communal values subdimension and post-materialist score were not associated with individuals’ *burden* view of older persons, regardless of the model specification.

**Table 4:** Odds ratio of logistic regressions of views towards older persons using aggregate subdimensions of personal value.

	Older people are a burden on society			Older people get more than fair share		
Variables	Singapore	Hong Kong	Japan	Singapore	Hong Kong	Japan
*Key explanatory variables*:						
Agentic values	1.339***	1.881***	1.958***	1.001	1.553***	1.317**
	(0.118)	(0.227)	(0.330)	(0.062)	(0.138)	(0.115)
Communal values	0.993	0.890	1.047	1.236**	0.991	0.990
	(0.112)	(0.118)	(0.168)	(0.093)	(0.098)	(0.081)
Post-materialist values	1.103	0.963	0.824	1.027	0.909	0.970
	(0.072)	(0.079)	(0.085)	(0.048)	(0.054)	(0.055)
*Socio-demographic controls*:						
Female	0.855	0.642*	0.792	0.757**	0.863	1.076
	(0.119)	(0.117)	(0.170)	(0.079)	(0.120)	(0.126)
Age bands (ref: above 60):						
18–30	0.731	0.455*	0.597	1.411	0.645	1.548
	(0.195)	(0.178)	(0.203)	(0.287)	(0.196)	(0.372)
31–40	0.765	0.342**	0.333**	1.611*	0.792	1.631**
	(0.198)	(0.116)	(0.121)	(0.319)	(0.206)	(0.289)
41–50	0.903	0.376**	0.306**	1.361	0.772	1.187
	(0.236)	(0.117)	(0.115)	(0.258)	(0.192)	(0.206)
51–60	0.673	0.600	0.328**	1.258	0.779	1.321
	(0.173)	(0.162)	(0.114)	(0.230)	(0.188)	(0.225)
Marital status (ref: single):						
Married/ Cohabiting	0.981	1.304	1.490	1.248	0.667*	1.124
	(0.172)	(0.351)	(0.433)	(0.177)	(0.131)	(0.205)
Others (divorced, widowed)	0.400*	1.730	1.534	1.443	0.532*	0.906
	(0.169)	(0.669)	(0.609)	(0.401)	(0.163)	(0.218)
Income	0.957	0.961	1.040	1.045	0.962	0.994
	(0.049)	(0.047)	(0.042)	(0.038)	(0.037)	(0.023)
Education	1.010	0.917	1.001	0.924**	0.905**	1.008
	(0.034)	(0.044)	(0.055)	(0.024)	(0.032)	(0.033)
Religious	0.963	0.726	0.701	0.919	0.974	0.867
	(0.140)	(0.171)	(0.166)	(0.096)	(0.161)	(0.109)
Currently employed	1.020	0.916	0.639*	1.182	0.942	1.346*
	(0.160)	(0.179)	(0.137)	(0.135)	(0.143)	(0.171)
Trust family	0.558***	0.645*	0.473***	0.737**	1.054	0.976
	(0.073)	(0.120)	(0.091)	(0.086)	(0.152)	(0.117)
Satisfied with life	0.864***	0.976	0.974	0.970	0.997	1.035
	(0.037)	(0.049)	(0.055)	(0.032)	(0.039)	(0.030)
Importance of government responsibility	0.964	1.018	0.958	0.979	0.975	0.943*
	(0.028)	(0.035)	(0.042)	(0.021)	(0.027)	(0.022)
*N =*	1,970	997	2,053	1,971	991	1,525
Pseudo R2 (adjusted)	0.054	0.106	0.105	0.023	0.042	0.036
Log likelihood	-829.3	-422.5	-421.6	-1322	-656.4	-994.8

*Notes*:

*** p<0.001,

** p<0.01,

* p<0.05.

Data is from WVS study wave 6 (2010–2014). Odds ratios from the logistic regressions are reported, together with the robust standard errors in parentheses. Individual-level weights are used in the analysis; see text. Results are based on the full model (model 3); regression results using intermediary models 1 and 2 are provided in the supplementary material (S2 and S3 Tables).

Other correlates of the *burden* view in the final model included age, female, martial status, trust family, and life satisfaction. Younger respondents in Japan and Hong Kong were significantly less likely to consider the older population as a burden compared to those more advanced in age. Women in Hong Kong were less likely to agree with the burden statement than men. Singaporean respondents who were divorced or widowed were less likely to view older persons as burden compared to those who were single, as were those more satisfied with their life. In all three societies—particularly Singapore and Japan—persons who trusted their family had significantly lower odds of perceiving older adults as burden.

Personal values similarly play a key role in explaining the *get more than fair share* outcome (Table 4, last three columns). The agentic values subdimension was positively associated with that perception in Japan (OR = 1.317, *p*<0.01) and Hong Kong (OR = 1.553, *p*<0.001). In Singapore, however, the communal values subdimension was significantly associated with such view instead (OR = 1.236, *p*<0.01). Respondents with higher educational attainment in Singapore and Hong Kong were less likely to agree with the view, while those aged 31–40 in Japan and Singapore were more likely to do so. Female, married, greater trust in family, and stronger belief in government responsibility were negatively related to the outcome.

### Logistic regressions using 10 disaggregated values

Table 5 displays the empirical results using the disaggregate configuration for personal values (i.e., 10 value types) instead of the aggregate subdimensions. These results based on the final model are analogous to those presented earlier in Table 4. Only the estimated ORs of the 10 value types are shown for brevity; full regression results are provided in online supplementary material (S4 Table). Three main observations are worth highlighting. First, this analysis reveals the primary driver(s) of the observed association between the subdimensions and the respective outcomes. For example, power (social status and wealth) was the key contributor of the positive association between the agentic values subdimension and the *burden* outcome in Singapore and Japan. By contrast, stimulation (excitement and novelty) was the key contributing component in Hong Kong PRC. Universalism values (concern for the welfare of those in the larger society) largely accounted for the positive association between the communal values subdimension and the *get more than fair share* outcome.

**Table 5:** Odds ratio of logistic regressions of views towards older persons using 10 disaggregate personal value types.

Variables	Older people are a burden on society			Older people get more than fair share		
	Singapore	Hong Kong	Japan	Singapore	Hong Kong	Japan
*Agentic values*:						
Power	1.402***	1.150	1.326*	1.061	1.109	1.144*
	(0.090)	(0.093)	(0.152)	(0.049)	(0.070)	(0.078)
Achievement	1.052	0.977	1.201	1.081	1.100	1.067
	(0.077)	(0.082)	(0.124)	(0.055)	(0.069)	(0.062)
Hedonism	0.907	0.988	1.080	0.916	0.991	1.042
	(0.065)	(0.083)	(0.112)	(0.044)	(0.059)	(0.056)
Stimulation	1.117	1.443***	1.047	0.968	1.118	1.000
	(0.081)	(0.104)	(0.126)	(0.047)	(0.068)	(0.063)
Self-direction	0.889	1.085	1.093	0.992	1.109	1.053
	(0.065)	(0.090)	(0.126)	(0.046)	(0.065)	(0.057)
*Communal values*:						
Security	0.920	1.302*	0.979	0.984	1.021	1.043
	(0.067)	(0.138)	(0.092)	(0.048)	(0.071)	(0.053)
Conformity	0.927	1.060	0.952	1.057	0.878*	1.104
	(0.072)	(0.084)	(0.108)	(0.055)	(0.054)	(0.063)
Tradition	1.117	0.920	1.025	1.054	1.014	0.950
	(0.081)	(0.068)	(0.105)	(0.051)	(0.055)	(0.049)
Universalism	1.163	1.048	0.950	1.144*	1.070	0.977
	(0.096)	(0.095)	(0.114)	(0.062)	(0.075)	(0.062)
Benevolence	0.903	0.748**	1.210	0.973	1.091	0.920
	(0.070)	(0.083)	(0.139)	(0.052)	(0.090)	(0.057)
*All other controls*	*Yes*	*Yes*	*Yes*	*Yes*	*Yes*	*Yes*
*N =*	1,970	997	2,053	1,971	991	1,525
Pseudo R2	0.077	0.134	0.111	0.028	0.049	0.041
Log likelihood	-809	-409	-419	-1315	-652	-990
Mean of dep var	0.163	0.183	0.061	0.443	0.524	0.409
SD of dep var	0.370	0.386	0.239	0.497	0.500	0.492

*Notes*:

*** p<0.001,

** p<0.01,

* p<0.05.

Data is from WVS study wave 6 (2010–2014). Odds ratios from the logistic regressions are reported, together with the robust standard errors in parentheses. Individual-level weights are used in the analysis; see text. Results are based on the full model (model 3). Other controls are not shown here for brevity; the full set of regression results are provided in the supplementary material (S4 Table).

Second, Hong Kongers with security-oriented values were 30% more likely to agree with the *burden* statement, while those with benevolence-oriented values were 25% less likely to do so. These significant effects approximately cancelled each other out; other components had ORs close to unity. This explains why the aggregate communion subdimension was not associated with the *burden* view. Third, R-squared values were systematically higher as compared to Table 4. For instance, the adjusted R-squared values for the *more than fair share* outcome were 0.028, 0.049, and 0.041 for Singapore, Hong Kong and Japan, respectively, in Table 5 (compare 0.023, 0.042, and 0.036 in Table 4). For the *burden* outcome, we also observed an improvement in the adjusted R-squared values from 0.054, 0.106, and 0.105 (Table 4) to 0.077, 0.134, and 0.111 (Table 5), respectively, for Singapore, Hong Kong, and Japan.

Thus far, our results are based on the full samples which include adults age 18 to as old as age 89. While this allows us to derive more generalizable results based on representative samples across a wide age spectrum, there may be some downward bias in how older adults respond to the ageist-related statements presented since they themselves are elderly. In some societies, the bias may also be upwards as suggested in our descriptive analysis indicating a higher prevalence of persons above age 60 agreeing with the *burden* statement. Given these concerns, we conducted sensitivity analyses by excluding older persons aged above 60 from the sample. Table 6 shows the empirical results based on the reduced sample. The key finding is that our main results are largely robust to this variation in sample. Specifically, the power and stimulation value types were statistically significant in explaining the relationship between agentic values and attitudes. The security and benevolence value types influenced the *burden* outcome in opposite directions.

**Table 6:** Sensitivity analysis: Odds ratio of logistic regressions using a reduced sample that excludes persons above age 60.

Variables	Older people are a burden on society			Older people get more than fair share		
	Singapore	Hong Kong	Japan	Singapore	Hong Kong	Japan
*Agentic values*:						
Power	1.417***	1.191	1.290	1.057	1.188*	1.198*
	(0.095)	(0.120)	(0.215)	(0.051)	(0.084)	(0.099)
Achievement	1.064	1.022	1.278	1.064	1.089	1.040
	(0.085)	(0.109)	(0.193)	(0.057)	(0.078)	(0.071)
Hedonism	0.930	0.920	1.116	0.917	0.996	0.989
	(0.073)	(0.101)	(0.160)	(0.048)	(0.067)	(0.063)
Stimulation	1.084	1.434***	1.112	0.985	1.093	0.994
	(0.086)	(0.127)	(0.180)	(0.051)	(0.074)	(0.073)
Self-direction	0.958	1.229	1.129	1.041	1.114	1.052
	(0.074)	(0.132)	(0.187)	(0.053)	(0.074)	(0.068)
*Communal values*:						
Security	0.846*	1.251	0.981	0.959	0.934	1.022
	(0.067)	(0.157)	(0.135)	(0.051)	(0.072)	(0.063)
Conformity	0.928	1.144	0.835	1.069	0.858*	1.089
	(0.078)	(0.121)	(0.138)	(0.059)	(0.060)	(0.075)
Tradition	1.157	0.957	1.129	1.081	1.018	0.939
	(0.091)	(0.090)	(0.178)	(0.056)	(0.062)	(0.060)
Universalism	1.168	0.980	0.766	1.133*	1.108	1.046
	(0.106)	(0.100)	(0.126)	(0.067)	(0.084)	(0.081)
Benevolence	0.949	0.733*	1.233	0.941	1.120	0.919
	(0.080)	(0.095)	(0.196)	(0.054)	(0.102)	(0.068)
*All other controls*	*Yes*	*Yes*	*Yes*	*Yes*	*Yes*	*Yes*
*N =*	1669	813	1351	1670	823	973
Pseudo R2	0.090	0.132	0.114	0.033	0.054	0.040
Log likelihood	-630	-303	-207	-1038	-540	-643
Mean of dep var	0.163	0.152	0.041	0.451	0.506	0.448
SD of dep var	0.369	0.359	0.198	0.498	0.500	0.498

*Notes*:

*** p<0.001,

** p<0.01,

* p<0.05.

Data is from WVS study wave 6 (2010–2014). Odds ratios from the logistic regressions are reported, together with the robust standard errors in parentheses. Individual-level weights are used in the analysis; see text. Results are based on the full model (model 3). Other controls are not shown here for brevity; the full set of regression results are provided in the supplementary material (S5 Table).

## Discussion

In this study, we examine the relationship between personal values and people’s attitudes toward older adults in three aging Asian societies. In addition to the aggregate measures of personal values (agency and communion subdimensions), we distinguished across 10 different value types based on Schwartz’s theory of values. The main finding from our study is that disaggregating personal value types provides greater prognostic power and more valuable insights on the relationship between values and attitudes than the conventionally-used aggregate measures. Moreover, we demonstrate that basic value types which are motivationally distinct may impact people’s views toward older adults differently, even if they theoretically belong to the same subdimension. In one of the samples analyzed, the Benevolence and Security value types within the communion subdimension influenced attitudes in opposite directions causing the effects to cancel each other out at the aggregate level. Consequently, analysis using only the aggregated measures can be misleading because the lack of association between the communion subdimension and attitudes leads one to believe that communitarian values do not predict people’s attitudes towards older adults, when they actually do. It is therefore important to acknowledge that the 10 different value types are fundamentally and motivationally distinct when investigating the relationship between people’s personal values and their attitudes.

Furthermore, the disaggregated analysis can provide administrators and policymakers with insights regarding ways to improve people’s attitudes towards older adults. As an example, our finding that the positive association between agentic values and attitudes was driven predominantly by power (social status and wealth) and stimulation (excitement and novelty) value types implies that interventions aimed at reducing ageist attitudes can potentially target individuals with agentic traits by emphasizing notions of power (e.g., older adults’ economic success) and stimulation (e.g., positive images of older adults learning new things). In other words, to help reduce ageist-related attitudes in aging societies, policy interventions using the mass media or other channels should showcase older adults’ financial and economic successes, their high standing in the workplace and/or community, as well as older adults’ willingness to learn new things, adventurous spirit, or other related aspects. This is broadly consistent with other research studies highlighting the importance of educational efforts on aging that can help reinforce positive views of elderly persons. For instance, Ragan and Bowen [36] showed that giving people accurate information about older people in conjunction with reinforcement for change can alter people’s negative attitudes associated with aging and ageism. Aside from educational interventions through videos or seminars, intergenerational extended contact (i.e., friends who had positive relations with older adults) can also facilitate improvements in attitudes toward older adults [9].

Our study was also informative about whether and how personal values influenced peoples’ attitudes towards older adults in the selected Asian Confucian societies. In all three economies, the agency subdimension was a more consistent predictor of unfavorable attitudes toward older adults than the communion subdimension. The significant role of agentic values can be rationalized since agency-oriented people predominantly serve self-interests, instead of collectivist interests as exemplified by the latent obligation to take care of older generations in one’s society. Also, the samples used included young and middle-aged adult respondents who tend to perceive achievement and social dominance as features of youth rather than old age [3, 10]. In terms of other correlates of attitudes toward older adults, we found age, sex, education, and trust in family to be particularly salient. Greater trust in family was associated with lower odds of perceiving older adults as burden across all samples. Furthermore, Singaporeans who trusted their family also had significantly lower odds of agreeing that older people get more than their fair share. Trust cements relationships by allowing people to live and work together, and this sense of belonging to a group may have contributed to more positive attitudes toward older adults in general. Among the more conventional factors, age and female were negatively related to the *burden* outcome. For the *more than fair share* outcome, it was noted that higher educational attainment reduced the odds of negative attitudes toward older adults. These findings are largely consistent with prior studies [11–13].

### Limitations and strengths

The present study implements a novel analytical approach in examining the relationship between personal values and people’s attitudes toward older adults. To the best of our knowledge, this is the first study to examine how each of the 10 motivationally distinct value types affects individuals’ attitudes toward older adults, rather than rely on the two aggregate measures. Other strengths of this study are its community-based representative population samples, comparative analysis across three aging (or aged) countries in Asia, and the adjustment for new potential covariates within the empirical framework. All the four additional control variables which we included in the regression are useful in explaining people’s unfavorable views towards older adults, albeit to different extents and in different contexts. Trust in family and being satisfied with life were strongly associated with the *burden* outcome, whereas being currently employed and stronger belief in government responsibility were only weakly associated with the *more than fair share* outcome.

Nonetheless, our study has a few limitations that future research can remedy. First, data on attitudes, personal values, and socio-economic factors (e.g., income and education) are self-reported and may be subject to imprecise measurement. For instance, there may be social desirability bias given that some respondents may under-report socially undesirable attitudes such as perceiving older adults as a burden to society [37]. Second, our cross-sectional dataset precludes us from analyzing changes in attitudes towards older adults over time, or whether measures of personal values remained stable over time. Finally, although we have controlled for many possible confounders in the multivariate regression framework, there may still be some omitted variables. For example, we are unable to include a household composition variable due to data limitation.

While our results provide some suggestive evidence that trust influences people’s attitudes towards older adults, this is primarily limited only to the trust in family variable we used. Future research is required to clarify the links between social capital and attitudes towards older adults, particularly given the possible reverse causality between trust levels and attitudes. Future research with more comprehensive datasets can also explore the respective roles of the personal values on attitudes in different geographical regions, how personal values relate to positive attitudes of older adults, as well as possible interactions among the distinct value types.

## Conclusions

Global population aging necessitates a better understanding of how individuals’ personal values shape their attitudes toward older adults. Our study provides the first empirical evidence about the relative importance of 10 different personal value types in predicting such attitudes, and demonstrates how the new insights generated from a disaggregated approach complements and supplements the conventional aggregate-level analysis using the agency and communion pairing. Viewing older adults as a burden on society or getting more than their fair share are ageist-related attitudes that can lead to wide-ranging consequences for society and for individuals’ health and well-being, including poorer physical and mental health and reduced quality of life for older persons. The findings of this study strengthen the case for educational efforts to combat ageism by constantly reinforcing positive images of elderly persons in public discourse and on social media, particularly by showcasing older adults’ financial and economic successes, high standing in society, adventurous spirit, as well as their willingness to learn new things.

## Supporting information

S1 Table Attitudes and personal values (aggregate) by age and sex. (DOCX)

S2 Table Odds ratio of hierarchical logistic regressions predicting the view that “older people are a burden on society”. (DOCX)

S3 Table Odds ratio of hierarchical logistic regressions predicting the view that “older people get more than their fair share”. (DOCX)

S4 Table Logistic regressions of views towards older persons using 10 disaggregate value variables (full regression results). (DOCX)

S5 Table Sensitivity analysis using a reduced sample that excludes persons above age 60 (full regression results). (DOCX)
