# Supplementary material for: Personal values and people’s attitudes toward older adults
Source: PLoS One. 2023 Aug 2;18(8):e0288589. doi: 10.1371/journal.pone.0288589 (PMC10395910; doi:10.1371/journal.pone.0288589)
Supplement: S1 Table — (DOCX) [file pone.0288589.s001.docx]

***Personal Values and People’s Attitudes Toward Older Adults (Supplementary material)***

**S1 Table. Attitudes and personal values (aggregate) by age and sex**

|  | **Singapore** | | **Hong Kong** | | **Japan** | |
| --- | --- | --- | --- | --- | --- | --- |
|  | **Mean** | **SD** | **Mean** | **SD** | **Mean** | **SD** |
|  | *Males only* | | | | | |
| Older people as a burden on society | 18.33% |  | 22.57% |  | 6.99% |  |
| Older people get more than their fair share | 48.07% |  | 55.68% |  | 41.69% |  |
| Agentic values | 3.90 | 0.93 | 3.66 | 0.85 | 2.74 | 0.70 |
| Communal values | 4.19 | 0.76 | 4.23 | 0.78 | 3.32 | 0.72 |
|  | *Females only* | | | | | |
| Older people as a burden on society | 14.84% |  | 14.68% |  | 5.23% |  |
| Older people get more than their fair share | 41.39% |  | 50.46% |  | 39.95% |  |
| Agentic values | 3.77 | 0.93 | 3.40 | 0.84 | 2.57 | 0.70 |
| Communal values | 4.25 | 0.74 | 4.27 | 0.76 | 3.46 | 0.76 |
|  | *Ages 18-30* | | | | | |
| Older people as a burden on society | 17.40% |  | 14.16% |  | 6.69% |  |
| Older people get more than their fair share | 41.66% |  | 50.46% |  | 46.24% |  |
| Agentic values | 4.05 | 0.84 | 3.73 | 0.78 | 3.07 | 0.77 |
| Communal values | 4.18 | 0.78 | 4.06 | 0.68 | 3.37 | 0.82 |
|  | *Ages 31-40* | | | | | |
| Older people as a burden on society | 15.49% |  | 11.68% |  | 3.75% |  |
| Older people get more than their fair share | 48.26% |  | 49.75% |  | 49.41% |  |
| Agentic values | 3.73 | 0.91 | 3.51 | 0.83 | 2.77 | 0.70 |
| Communal values | 4.20 | 0.80 | 4.23 | 0.72 | 3.31 | 0.71 |
|  | *Ages 41-50* | | | | | |
| Older people as a burden on society | 17.29% |  | 13.43% |  | 3.42% |  |
| Older people get more than their fair share | 45.46% |  | 49.07% |  | 41.86% |  |
| Agentic values | 3.70 | 0.95 | 3.43 | 0.84 | 2.68 | 0.68 |
| Communal values | 4.18 | 0.76 | 4.23 | 0.78 | 3.37 | 0.70 |
|  | *Ages 51-60* | | | | | |
| Older people as a burden on society | 14.35% |  | 21.76% |  | 3.19% |  |
| Older people get more than their fair share | 47.05% |  | 53.37% |  | 42.56% |  |
| Agentic values | 3.79 | 0.88 | 3.51 | 0.89 | 2.49 | 0.62 |
| Communal values | 4.29 | 0.72 | 4.29 | 0.85 | 3.31 | 0.67 |
|  | *Age above 60* | | | | | |
| Older people as a burden on society | 16.59% |  | 33.14% |  | 10.16% |  |
| Older people get more than their fair share | 40.91% |  | 61.08% |  | 33.88% |  |
| Agentic values | 3.77 | 1.05 | 3.40 | 0.89 | 2.51 | 0.65 |
| Communal values | 4.29 | 0.70 | 4.50 | 0.77 | 3.50 | 0.78 |
| *N (full sample) =* | *1972* | | *1000* | | *2443* | |

*Notes*: Data is from WVS study wave 6 (2010-2014). Percentages shown for categorical variables; means and standard deviations shown for continuous variables. Individual-level weights are applied.
