# Supplementary material for: Personal values and people’s attitudes toward older adults
Source: PLoS One. 2023 Aug 2;18(8):e0288589. doi: 10.1371/journal.pone.0288589 (PMC10395910; doi:10.1371/journal.pone.0288589)
Supplement: S2 Table — (DOCX) [file pone.0288589.s002.docx]

***Personal Values and People’s Attitudes Toward Older Adults (Supplementary material)***

**S2 Table. Odds ratio of hierarchical logistic regressions predicting the view that “older people are a burden on society”**

| **Variables** | **Singapore** | | | **Hong Kong** | | | **Japan** | | |
| --- | --- | --- | --- | --- | --- | --- | --- | --- | --- |
|  | **Model 1** | **Model 2** | **Model 3** | **Model 1** | **Model 2** | **Model 3** | **Model 1** | **Model 2** | **Model 3** |
| *Key explanatory variables:* |  |  |  |  |  |  |  |  |  |
| Agentic values | 1.308** | 1.367*** | 1.339*** | 1.792*** | 1.830*** | 1.881*** | 1.920*** | 1.916*** | 1.958*** |
|  | (0.108) | (0.119) | (0.118) | (0.218) | (0.220) | (0.227) | (0.314) | (0.320) | (0.330) |
| Communal values | 0.969 | 0.981 | 0.993 | 0.875 | 0.886 | 0.890 | 0.965 | 0.999 | 1.047 |
|  | (0.104) | (0.109) | (0.112) | (0.112) | (0.116) | (0.118) | (0.151) | (0.159) | (0.168) |
| Post-materialist values | 1.118 | 1.120 | 1.103 | 0.948 | 0.976 | 0.963 | 0.837 | 0.848 | 0.824 |
|  | (0.071) | (0.071) | (0.072) | (0.074) | (0.079) | (0.079) | (0.086) | (0.086) | (0.085) |
| *Socio-demographic controls:* |  |  |  |  |  |  |  |  |  |
| Female | 0.804 | 0.832 | 0.855 | 0.715 | 0.655* | 0.642* | 0.858 | 0.854 | 0.792 |
|  | (0.108) | (0.112) | (0.119) | (0.127) | (0.116) | (0.117) | (0.170) | (0.177) | (0.170) |
| Age bands (ref: above 60): |  |  |  |  |  |  |  |  |  |
| 18-30 | 0.946 | 0.834 | 0.731 | 0.264*** | 0.474 | 0.455* | 0.464** | 0.548 | 0.597 |
|  | (0.190) | (0.218) | (0.195) | (0.078) | (0.182) | (0.178) | (0.130) | (0.176) | (0.203) |
| 31-40 | 0.911 | 0.872 | 0.765 | 0.242*** | 0.365** | 0.342** | 0.298*** | 0.303*** | 0.333** |
|  | (0.204) | (0.219) | (0.198) | (0.071) | (0.118) | (0.116) | (0.095) | (0.102) | (0.121) |
| 41-50 | 0.970 | 0.960 | 0.903 | 0.285*** | 0.375*** | 0.376** | 0.283*** | 0.276*** | 0.306** |
|  | (0.224) | (0.236) | (0.236) | (0.079) | (0.111) | (0.117) | (0.096) | (0.096) | (0.115) |
| 51-60 | 0.831 | 0.808 | 0.673 | 0.513** | 0.595* | 0.600 | 0.303*** | 0.292*** | 0.328** |
|  | (0.200) | (0.195) | (0.173) | (0.128) | (0.154) | (0.162) | (0.093) | (0.090) | (0.114) |
| Marital status (ref: single): |  |  |  |  |  |  |  |  |  |
| Married/ Cohabiting |  | 0.944 | 0.981 |  | 1.223 | 1.304 |  | 1.243 | 1.490 |
|  |  | (0.165) | (0.172) |  | (0.331) | (0.351) |  | (0.353) | (0.433) |
| Others (divorced, widowed) |  | 0.436* | 0.400* |  | 1.670 | 1.730 |  | 1.407 | 1.534 |
|  |  | (0.178) | (0.169) |  | (0.637) | (0.669) |  | (0.547) | (0.609) |
| Income |  | 0.907* | 0.957 |  | 0.946 | 0.961 |  | 1.026 | 1.040 |
|  |  | (0.044) | (0.049) |  | (0.044) | (0.047) |  | (0.040) | (0.042) |
| Education |  | 0.999 | 1.010 |  | 0.901* | 0.917 |  | 0.986 | 1.001 |
|  |  | (0.032) | (0.034) |  | (0.043) | (0.044) |  | (0.054) | (0.055) |
| Religious |  | 0.873 | 0.963 |  | 0.736 | 0.726 |  | 0.684 | 0.701 |
|  |  | (0.123) | (0.140) |  | (0.169) | (0.171) |  | (0.159) | (0.166) |
| Currently employed |  |  | 1.020 |  |  | 0.916 |  |  | 0.639* |
|  |  |  | (0.160) |  |  | (0.179) |  |  | (0.137) |
| Trust family |  |  | 0.558*** |  |  | 0.645* |  |  | 0.473*** |
|  |  |  | (0.073) |  |  | (0.120) |  |  | (0.091) |
| Satisfied with life |  |  | 0.864*** |  |  | 0.976 |  |  | 0.974 |
|  |  |  | (0.037) |  |  | (0.049) |  |  | (0.055) |
| Importance of government responsibility |  |  | 0.964 |  |  | 1.018 |  |  | 0.958 |
|  |  |  | (0.028) |  |  | (0.035) |  |  | (0.042) |
|  |  |  |  |  |  |  |  |  |  |
| *N=* | 1,970 | 1,969 | 1,969 | 997 | 997 | 990 | 2,053 | 2,053 | 2,053 |
| Pseudo R2 | 0.0196 | 0.0275 | 0.0542 | 0.0790 | 0.0937 | 0.106 | 0.0659 | 0.0736 | 0.105 |
| Log likelihood | -859.9 | -852.7 | -829.3 | -436.4 | -429.4 | -422.5 | -439.9 | -436.3 | -421.6 |

*Notes*: *** p<0.001, ** p<0.01, * p<0.05. Data is from WVS study wave 6 (2010-2014). Odds ratios from the logistic regressions are reported, together with the robust standard errors in parentheses. Individual-level weights are used in the analysis; see text.
