# Supplementary material for: Personal values and people’s attitudes toward older adults
Source: PLoS One. 2023 Aug 2;18(8):e0288589. doi: 10.1371/journal.pone.0288589 (PMC10395910; doi:10.1371/journal.pone.0288589)
Supplement: S3 Table — (DOCX) [file pone.0288589.s003.docx]

***Personal Values and People’s Attitudes Toward Older Adults (Supplementary material)***

**S3 Table. Odds ratio of hierarchical logistic regressions predicting the view that “older people get more than their fair share”**

| **Variables** | **Singapore** | | | **Hong Kong** | | | **Japan** | | |
| --- | --- | --- | --- | --- | --- | --- | --- | --- | --- |
|  | **Model 1** | **Model 2** | **Model 3** | **Model 1** | **Model 2** | **Model 3** | **Model 1** | **Model 2** | **Model 3** |
| *Key explanatory variables:* |  |  |  |  |  |  |  |  |  |
| Agentic values | 1.007 | 1.010 | 1.001 | 1.526*** | 1.550*** | 1.553*** | 1.348*** | 1.347*** | 1.317** |
|  | (0.060) | (0.062) | (0.062) | (0.132) | (0.137) | (0.138) | (0.116) | (0.117) | (0.115) |
| Communal values | 1.210** | 1.226** | 1.236** | 0.966 | 0.991 | 0.991 | 0.943 | 0.961 | 0.990 |
|  | (0.088) | (0.091) | (0.093) | (0.091) | (0.096) | (0.098) | (0.074) | (0.077) | (0.081) |
| Post-materialist values | 1.029 | 1.031 | 1.027 | 0.892* | 0.908 | 0.909 | 0.970 | 0.972 | 0.970 |
|  | (0.047) | (0.047) | (0.048) | (0.052) | (0.054) | (0.054) | (0.055) | (0.055) | (0.055) |
| *Socio-demographic controls:* |  |  |  |  |  |  |  |  |  |
| Female | 0.753** | 0.730** | 0.757** | 0.888 | 0.875 | 0.863 | 0.975 | 1.011 | 1.076 |
|  | (0.075) | (0.074) | (0.079) | (0.118) | (0.119) | (0.120) | (0.107) | (0.113) | (0.126) |
| Age bands (ref: above 60): |  |  |  |  |  |  |  |  |  |
| 18-30 | 1.010 | 1.497* | 1.411 | 0.612* | 0.621 | 0.645 | 1.448* | 1.604* | 1.548 |
|  | (0.154) | (0.301) | (0.287) | (0.140) | (0.185) | (0.196) | (0.270) | (0.378) | (0.372) |
| 31-40 | 1.341 | 1.792** | 1.611* | 0.619* | 0.760 | 0.792 | 1.797*** | 1.771*** | 1.631** |
|  | (0.223) | (0.343) | (0.319) | (0.137) | (0.187) | (0.206) | (0.284) | (0.296) | (0.289) |
| 41-50 | 1.222 | 1.480* | 1.361 | 0.640* | 0.748 | 0.772 | 1.357 | 1.308 | 1.187 |
|  | (0.206) | (0.269) | (0.258) | (0.141) | (0.175) | (0.192) | (0.215) | (0.214) | (0.206) |
| 51-60 | 1.266 | 1.390 | 1.258 | 0.703 | 0.767 | 0.779 | 1.464* | 1.417* | 1.321 |
|  | (0.219) | (0.247) | (0.230) | (0.157) | (0.177) | (0.188) | (0.223) | (0.225) | (0.225) |
| Marital status (ref: single): |  |  |  |  |  |  |  |  |  |
| Married/ Cohabiting |  | 1.229 | 1.248 |  | 0.673* | 0.667* |  | 1.150 | 1.124 |
|  |  | (0.175) | (0.177) |  | (0.133) | (0.131) |  | (0.205) | (0.205) |
| Others (divorced, widowed) |  | 1.495 | 1.443 |  | 0.535* | 0.532* |  | 0.956 | 0.906 |
|  |  | (0.408) | (0.401) |  | (0.164) | (0.163) |  | (0.229) | (0.218) |
| Income |  | 1.030 | 1.045 |  | 0.964 | 0.962 |  | 1.005 | 0.994 |
|  |  | (0.036) | (0.038) |  | (0.035) | (0.037) |  | (0.022) | (0.023) |
| Education |  | 0.928** | 0.924** |  | 0.905** | 0.905** |  | 1.012 | 1.008 |
|  |  | (0.023) | (0.024) |  | (0.031) | (0.032) |  | (0.033) | (0.033) |
| Religious |  | 0.891 | 0.919 |  | 0.974 | 0.974 |  | 0.872 | 0.867 |
|  |  | (0.092) | (0.096) |  | (0.161) | (0.161) |  | (0.109) | (0.109) |
| Currently employed |  |  | 1.182 |  |  | 0.942 |  |  | 1.346* |
|  |  |  | (0.135) |  |  | (0.143) |  |  | (0.171) |
| Trust family |  |  | 0.737** |  |  | 1.054 |  |  | 0.976 |
|  |  |  | (0.086) |  |  | (0.152) |  |  | (0.117) |
| Satisfied with life |  |  | 0.970 |  |  | 0.997 |  |  | 1.035 |
|  |  |  | (0.032) |  |  | (0.039) |  |  | (0.030) |
| Importance of government responsibility |  |  | 0.979 |  |  | 0.975 |  |  | 0.943* |
|  |  |  | (0.021) |  |  | (0.027) |  |  | (0.022) |
|  |  |  |  |  |  |  |  |  |  |
| *N=* | 1,971 | 1,971 | 1,971 | 991 | 991 | 991 | 1,525 | 1,525 | 1,525 |
| Pseudo R2 | 0.0106 | 0.0172 | 0.0228 | 0.0310 | 0.0419 | 0.0429 | 0.0199 | 0.0235 | 0.0355 |
| Log likelihood | -1338 | -1330 | -1322 | -664.5 | -657 | -656.4 | -1011 | -1007 | -994.8 |

*Notes*: *** p<0.001, ** p<0.01, * p<0.05. Data is from WVS study wave 6 (2010-2014). Odds ratios from the logistic regressions are reported, together with the robust standard errors in parentheses. Individual-level weights are used in the analysis; see text.
