# Supplementary material for: Personal values and people’s attitudes toward older adults
Source: PLoS One. 2023 Aug 2;18(8):e0288589. doi: 10.1371/journal.pone.0288589 (PMC10395910; doi:10.1371/journal.pone.0288589)
Supplement: S4 Table — (DOCX) [file pone.0288589.s004.docx]

***Personal Values and People’s Attitudes Toward Older Adults (Supplementary material)***

**S4 Table. Logistic regressions of views towards older persons using 10 disaggregate value variables (full regression results)**

|  | **Older people are a burden on society** | | |  | **Older people get more than fair share** | | |
| --- | --- | --- | --- | --- | --- | --- | --- |
| Variables | **Singapore** | **Hong Kong** | **Japan** |  | **Singapore** | **Hong Kong** | **Japan** |
| *Agentic values:* |  |  |  |  |  |  |  |
| Power | 1.402*** | 1.150 | 1.326* |  | 1.061 | 1.109 | 1.144* |
|  | (0.090) | (0.093) | (0.152) |  | (0.049) | (0.070) | (0.078) |
| Achievement | 1.052 | 0.977 | 1.201 |  | 1.081 | 1.100 | 1.067 |
|  | (0.077) | (0.082) | (0.124) |  | (0.055) | (0.069) | (0.062) |
| Hedonism | 0.907 | 0.988 | 1.080 |  | 0.916 | 0.991 | 1.042 |
|  | (0.065) | (0.083) | (0.112) |  | (0.044) | (0.059) | (0.056) |
| Stimulation | 1.117 | 1.443*** | 1.047 |  | 0.968 | 1.118 | 1.000 |
|  | (0.081) | (0.104) | (0.126) |  | (0.047) | (0.068) | (0.063) |
| Self-direction | 0.889 | 1.085 | 1.093 |  | 0.992 | 1.109 | 1.053 |
|  | (0.065) | (0.090) | (0.126) |  | (0.046) | (0.065) | (0.057) |
| *Communal values:* |  |  |  |  |  |  |  |
| Security | 0.920 | 1.302* | 0.979 |  | 0.984 | 1.021 | 1.043 |
|  | (0.067) | (0.138) | (0.092) |  | (0.048) | (0.071) | (0.053) |
| Conformity | 0.927 | 1.060 | 0.952 |  | 1.057 | 0.878* | 1.104 |
|  | (0.072) | (0.084) | (0.108) |  | (0.055) | (0.054) | (0.063) |
| Tradition | 1.117 | 0.920 | 1.025 |  | 1.054 | 1.014 | 0.950 |
|  | (0.081) | (0.068) | (0.105) |  | (0.051) | (0.055) | (0.049) |
| Universalism | 1.163 | 1.048 | 0.950 |  | 1.144* | 1.070 | 0.977 |
|  | (0.096) | (0.095) | (0.114) |  | (0.062) | (0.075) | (0.062) |
| Benevolence | 0.903 | 0.748** | 1.210 |  | 0.973 | 1.091 | 0.920 |
|  | (0.070) | (0.083) | (0.139) |  | (0.052) | (0.090) | (0.057) |
|  | | | |  |  |  |  |
| *Socio-demographic controls:* | | | |  |  |  |  |
| Female | 0.856 | 0.707 | 0.770 |  | 0.768* | 0.875 | 1.083 |
|  | (0.121) | (0.133) | (0.166) |  | (0.081) | (0.125) | (0.129) |
| Age bands (ref: above 60): | |  |  |  |  |  |  |
| 18-30 | 0.740 | 0.398* | 0.580 |  | 1.438 | 0.667 | 1.558 |
|  | (0.203) | (0.161) | (0.201) |  | (0.295) | (0.207) | (0.385) |
| 31-40 | 0.748 | 0.290*** | 0.333** |  | 1.627* | 0.805 | 1.548* |
|  | (0.203) | (0.101) | (0.126) |  | (0.324) | (0.214) | (0.285) |
| 41-50 | 0.848 | 0.314*** | 0.308** |  | 1.348 | 0.784 | 1.181 |
|  | (0.226) | (0.101) | (0.118) |  | (0.256) | (0.198) | (0.209) |
| 51-60 | 0.662 | 0.553* | 0.326** |  | 1.276 | 0.758 | 1.361 |
|  | (0.173) | (0.154) | (0.112) |  | (0.235) | (0.186) | (0.233) |
| Marital status (ref: single): | |  |  |  |  |  |  |
| Married | 0.955 | 1.370 | 1.445 |  | 1.241 | 0.653* | 1.107 |
|  | (0.170) | (0.368) | (0.425) |  | (0.176) | (0.129) | (0.203) |
| Others | 0.388* | 2.066 | 1.486 |  | 1.433 | 0.538* | 0.897 |
|  | (0.165) | (0.811) | (0.598) |  | (0.404) | (0.167) | (0.218) |
| Income | 0.960 | 0.955 | 1.037 |  | 1.050 | 0.970 | 0.988 |
|  | (0.047) | (0.049) | (0.043) |  | (0.038) | (0.038) | (0.023) |
| Education | 1.012 | 0.920 | 0.997 |  | 0.917*** | 0.910** | 1.001 |
|  | (0.035) | (0.044) | (0.057) |  | (0.024) | (0.033) | (0.033) |
| *Other controls:* |  |  |  |  |  |  |  |
| Post-materialist values | 1.136 | 0.969 | 0.820 |  | 1.026 | 0.914 | 0.996 |
|  | (0.076) | (0.081) | (0.086) |  | (0.048) | (0.055) | (0.058) |
| Religious | 0.920 | 0.770 | 0.674 |  | 0.910 | 0.937 | 0.905 |
|  | (0.137) | (0.188) | (0.163) |  | (0.096) | (0.158) | (0.116) |
| Currently employed | 1.033 | 0.941 | 0.640* |  | 1.197 | 0.921 | 1.338* |
|  | (0.165) | (0.186) | (0.138) |  | (0.137) | (0.142) | (0.171) |
| Trust family | 0.562*** | 0.729 | 0.471*** |  | 0.734** | 1.036 | 0.985 |
|  | (0.078) | (0.138) | (0.094) |  | (0.086) | (0.154) | (0.119) |
| Satisfied with life | 0.878** | 1.001 | 0.965 |  | 0.976 | 0.999 | 1.043 |
|  | (0.037) | (0.052) | (0.056) |  | (0.033) | (0.039) | (0.031) |
| Importance of govt responsibility | 0.966 | 1.040 | 0.956 |  | 0.980 | 0.975 | 0.941** |
|  | (0.028) | (0.037) | (0.042) |  | (0.021) | (0.027) | (0.022) |
|  |  |  |  |  |  |  |  |
| *N=* | 1,970 | 997 | 2,053 |  | 1,971 | 991 | 1,525 |
| Pseudo R2 | 0.077 | 0.134 | 0.111 |  | 0.028 | 0.049 | 0.041 |
| Log likelihood | -809 | -409 | -419 |  | -1315 | -652 | -990 |
| Mean of dep var | 0.163 | 0.183 | 0.061 |  | 0.443 | 0.524 | 0.409 |
| SD of dep var | 0.370 | 0.386 | 0.239 |  | 0.497 | 0.500 | 0.492 |

*Notes*: *** p<0.001, ** p<0.01, * p<0.05. Data is from WVS study wave 6 (2010-2014). Odds ratios from the logistic regressions are reported, together with the robust standard errors in parentheses. Individual-level weights are used in the analysis; see text. Results are based on the full model (model 3).
