# Supplementary material for: Personal values and people’s attitudes toward older adults
Source: PLoS One. 2023 Aug 2;18(8):e0288589. doi: 10.1371/journal.pone.0288589 (PMC10395910; doi:10.1371/journal.pone.0288589)
Supplement: S5 Table — (DOCX) [file pone.0288589.s005.docx]

***Personal Values and People’s Attitudes Toward Older Adults (Supplementary material)***

**S5 Table. Sensitivity analysis using a reduced sample that excludes persons above age 60 (full regression results)**

|  | **Older people are a burden on society** | | |  | **Older people get more than fair share** | | |
| --- | --- | --- | --- | --- | --- | --- | --- |
| Variables | **Singapore** | **Hong Kong** | **Japan** |  | **Singapore** | **Hong Kong** | **Japan** |
| *Agentic values:* |  |  |  |  |  |  |  |
| Power | 1.417*** | 1.191 | 1.290 |  | 1.057 | 1.188* | 1.198* |
|  | (0.095) | (0.120) | (0.215) |  | (0.051) | (0.084) | (0.099) |
| Achievement | 1.064 | 1.022 | 1.278 |  | 1.064 | 1.089 | 1.040 |
|  | (0.085) | (0.109) | (0.193) |  | (0.057) | (0.078) | (0.071) |
| Hedonism | 0.930 | 0.920 | 1.116 |  | 0.917 | 0.996 | 0.989 |
|  | (0.073) | (0.101) | (0.160) |  | (0.048) | (0.067) | (0.063) |
| Stimulation | 1.084 | 1.434*** | 1.112 |  | 0.985 | 1.093 | 0.994 |
|  | (0.086) | (0.127) | (0.180) |  | (0.051) | (0.074) | (0.073) |
| Self-direction | 0.958 | 1.229 | 1.129 |  | 1.041 | 1.114 | 1.052 |
|  | (0.074) | (0.132) | (0.187) |  | (0.053) | (0.074) | (0.068) |
| *Communal values:* |  |  |  |  |  |  |  |
| Security | 0.846* | 1.251 | 0.981 |  | 0.959 | 0.934 | 1.022 |
|  | (0.067) | (0.157) | (0.135) |  | (0.051) | (0.072) | (0.063) |
| Conformity | 0.928 | 1.144 | 0.835 |  | 1.069 | 0.858* | 1.089 |
|  | (0.078) | (0.121) | (0.138) |  | (0.059) | (0.060) | (0.075) |
| Tradition | 1.157 | 0.957 | 1.129 |  | 1.081 | 1.018 | 0.939 |
|  | (0.091) | (0.090) | (0.178) |  | (0.056) | (0.062) | (0.060) |
| Universalism | 1.168 | 0.980 | 0.766 |  | 1.133* | 1.108 | 1.046 |
|  | (0.106) | (0.100) | (0.126) |  | (0.067) | (0.084) | (0.081) |
| Benevolence | 0.949 | 0.733* | 1.233 |  | 0.941 | 1.120 | 0.919 |
|  | (0.080) | (0.095) | (0.196) |  | (0.054) | (0.102) | (0.068) |
|  | | | |  |  |  |  |
| *Socio-demographic controls:* | | | |  |  |  |  |
| Female | 0.952 | 0.832 | 0.713 |  | 0.781* | 0.867 | 0.980 |
|  | (0.146) | (0.184) | (0.240) |  | (0.088) | (0.139) | (0.145) |
| Age bands (ref: 51-60): | |  |  |  |  |  |  |
| 18-30 | 1.088 | 0.583 | 1.723 |  | 1.121 | 0.914 | 1.246 |
|  | (0.301) | (0.231) | (0.635) |  | (0.219) | (0.267) | (0.320) |
| 31-40 | 1.140 | 0.451* | 1.073 |  | 1.344 | 1.098 | 1.186 |
|  | (0.295) | (0.149) | (0.447) |  | (0.243) | (0.252) | (0.226) |
| 41-50 | 1.285 | 0.513* | 1.026 |  | 1.086 | 1.070 | 0.891 |
|  | (0.334) | (0.155) | (0.446) |  | (0.192) | (0.231) | (0.162) |
| Marital status (ref: single): | |  |  |  |  |  |  |
| Married | 0.947 | 1.141 | 1.851* |  | 1.155 | 0.653* | 1.214 |
|  | (0.181) | (0.346) | (0.575) |  | (0.172) | (0.140) | (0.244) |
| Others | 0.226* | 2.586 | 1.397 |  | 1.163 | 0.627 | 0.988 |
|  | (0.134) | (1.308) | (0.969) |  | (0.397) | (0.239) | (0.306) |
| Income | 0.941 | 1.006 | 1.015 |  | 1.019 | 0.983 | 0.988 |
|  | (0.049) | (0.067) | (0.061) |  | (0.040) | (0.045) | (0.028) |
| Education | 1.021 | 0.944 | 1.120 |  | 0.896*** | 0.914* | 1.024 |
|  | (0.039) | (0.054) | (0.131) |  | (0.025) | (0.036) | (0.046) |
| *Other controls:* |  |  |  |  |  |  |  |
| Post-materialist values | 1.120 | 0.887 | 0.706* |  | 1.043 | 0.877 | 1.093 |
|  | (0.079) | (0.085) | (0.112) |  | (0.052) | (0.059) | (0.079) |
| Religious | 0.966 | 0.805 | 0.588 |  | 0.962 | 1.004 | 0.852 |
|  | (0.155) | (0.224) | (0.238) |  | (0.109) | (0.188) | (0.141) |
| Currently employed | 0.955 | 1.140 | 0.554 |  | 1.178 | 0.853 | 1.450* |
|  | (0.158) | (0.255) | (0.185) |  | (0.145) | (0.139) | (0.255) |
| Trust family | 0.519*** | 0.641 | 0.596 |  | 0.684** | 1.089 | 0.848 |
|  | (0.078) | (0.150) | (0.169) |  | (0.087) | (0.185) | (0.125) |
| Satisfied with life | 0.889* | 0.925 | 0.926 |  | 0.996 | 0.995 | 1.013 |
|  | (0.042) | (0.062) | (0.073) |  | (0.036) | (0.046) | (0.038) |
| Importance of govt responsibility | 0.942 | 0.993 | 0.922 |  | 0.969 | 0.984 | 0.937* |
|  | (0.030) | (0.045) | (0.063) |  | (0.023) | (0.031) | (0.029) |
|  |  |  |  |  |  |  |  |
| *N=* | 1669 | 813 | 1351 |  | 1670 | 823 | 973 |
| Pseudo R2 | 0.090 | 0.132 | 0.114 |  | 0.033 | 0.054 | 0.040 |
| Log likelihood | -630 | -303 | -207 |  | -1038 | -540 | -643 |
| Mean of dep var | 0.163 | 0.152 | 0.041 |  | 0.451 | 0.506 | 0.448 |
| SD of dep var | 0.369 | 0.359 | 0.198 |  | 0.498 | 0.500 | 0.498 |

*Notes*: *** p<0.001, ** p<0.01, * p<0.05. Data is from WVS study wave 6 (2010-2014). Odds ratios from the logistic regressions are reported, together with the robust standard errors in parentheses. Individual-level weights are used in the analysis; see text. Results are based on the full model (model 3).
